# Challenging muscle homeostasis uncovers novel chaperone interactions in *Caenorhabditis elegans*

**DOI:** 10.3389/fmolb.2014.00021

**Published:** 2014-11-06

**Authors:** Anna Frumkin, Shiran Dror, Wojciech Pokrzywa, Yael Bar-Lavan, Ido Karady, Thorsten Hoppe, Anat Ben-Zvi

**Affiliations:** ^1^Department of Life Sciences and The National Institute for Biotechnology in the Negev, Ben-Gurion University of the NegevBeer Sheva, Israel; ^2^Institute for Genetics and Cologne Excellence Cluster on Cellular Stress Responses in Aging-Associated Diseases (CECAD), University of CologneCologne, Germany; ^3^Cologne Excellence Cluster on Cellular Stress Responses in Aging-Associated Diseases, CECAD Research Center, University of CologneCologne, Germany

**Keywords:** *Caenorhabditis elegans*, chaperones, DAF-21, Hsp90, misfolding, myosin, proteostasis, UNC-45

## Abstract

Proteome stability is central to cellular function and the lifespan of an organism. This is apparent in muscle cells, where incorrect folding and assembly of the sarcomere contributes to disease and aging. Apart from the myosin-assembly factor UNC-45, the complete network of chaperones involved in assembly and maintenance of muscle tissue is currently unknown. To identify additional factors required for sarcomere quality control, we performed genetic screens based on suppressed or synthetic motility defects in *Caenorhabditis elegans*. In addition to ethyl methyl sulfonate-based mutagenesis, we employed RNAi-mediated knockdown of candidate chaperones in *unc-45* temperature-sensitive mutants and screened for impaired movement at permissive conditions. This approach confirmed the cooperation between UNC-45 and Hsp90. Moreover, the screens identified three novel co-chaperones, CeHop (STI-1), CeAha1 (C01G10.8) and Cep23 (ZC395.10), required for muscle integrity. The specific identification of Hsp90 and Hsp90 co-chaperones highlights the physiological role of Hsp90 in myosin folding. Our work thus provides a clear example of how a combination of mild perturbations to the proteostasis network can uncover specific quality control modules.

## Introduction

The term molecular chaperone was first coined to describe the transient involvement of the nuclear protein nucleoplasmin in the assembly of the nucleosome subunits (Laskey et al., 1978). The concept of “assisting proteins” was then adopted to define a group of highly conserved proteins, some of which are heat shock proteins (HSPs), found to be transiently involved in polypeptide folding and in the assembly of various protein complexes, without being part of the final structure (Pelham, 1986; Ellis, 1987; Hemmingsen et al., 1988).

*In vitro* studies using purified components established a role for molecular chaperones in preventing aggregation and in the unfolding and reactivation of denatured proteins (Li et al., 2011; Bardwell and Jakob, 2012; Mattoo and Goloubinoff, 2014). For example, denatured model substrates, such as malate dehydrogenase, rhodanese and luciferase, were used in structure-function studies to elucidate chaperone activity, as a proxies for correct protein folding (Goloubinoff et al., 1989; Buchner et al., 1991; Martin et al., 1991; Schroder et al., 1993). In contrast, *in vivo* studies have been hampered by a lack of phenotypes that can be readily monitored. This challenge is further amplified when one considers that a single substrate is able to interact with many different chaperones within the cell.

Identifying specialized chaperones required for muscle assembly and maintenance offers a prime example of the difficulties associated with studying protein folding *in vivo* (Kim et al., 2008; Willis et al., 2009; Willis and Patterson, 2010). Although myosin subunits can spontaneously assemble into thick, filament-like structures *in vitro*, the myosin motor domain does not fold, assemble or function in an autonomous manner *in vivo* (Barral and Epstein, 1999; Srikakulam and Winkelmann, 2004). Thus far, screening for detectable phenotypes, such as those related to motility, muscle elongation and myofilament organization in *Caenorhabditis elegans*, identified only two chaperones, namely UNC-45 and UNC-23, among ~200 proteins required for sarcomere assembly, maintenance or function (Meissner et al., 2009; Benian and Epstein, 2011). While biochemical analysis of UNC-45-based interactions identified the molecular chaperone Hsp90 (Barral et al., 2002), knockdown or mutations of *C. elegans* Hsp90 (*daf-21*) results in only a minor effect on motility (Gaiser et al., 2011). This gap between phenotype and function might result from redundant functions of the proteostasis network, compensating for monogenic defects (Balch et al., 2008; Sawyer et al., 2011).

To study tissue-specific chaperone networks in a multicellular organism, we focused on the essential role of the myosin chaperone UNC-45, a member of the conserved UCS (UNC-45/CRO1/She4p) family, with the aim of uncovering novel chaperones involved in myosin folding. Temperature-sensitive (*ts*) mutants of *unc-45* result in motility and sarcomere organization defects at restrictive conditions but have no apparent phenotype at the permissive growth temperature of 15°C. *unc-45-associated* phenotypes can be exposed under permissive conditions or masked under restrictive conditions by genetic or chemical remodeling of proteostasis (Gidalevitz et al., 2006, 2009; Alavez et al., 2011; Gazda et al., 2013; Melkani et al., 2013; Feldman et al., 2014). Thus, *unc-45(ts)* can be viewed as a sensitized background to screen for molecular chaperones specifically associated with muscle function.

Here, we used a non-biased mutagenesis screen to identify chaperones that ameliorate *unc-45(ts)* phenotypes at restrictive conditions and targeted RNAi synthetic interaction screens to identify chaperones that aggravate *unc-45(ts)* phenotypes under permissive conditions. Using an epistatic analysis approach, we genetically verified the interaction between UNC-45 and Hsp90. Moreover, we identified the Hsp90 co-chaperones CeHop (STI-1), CeAha1 (C01G10.8), and Cep23 (ZC395.10) as novel and specific modulators of UNC-45-Hsp90 myosin-associated functions.

## Materials and methods

### Nematodes and growth conditions

*C. elegans* strains Bristol N2 (wild-type), DR94 (*unc-45(m94)*), CB286 (*unc-45(e286)*), RW1596 (*myo-3(st386)*; *stEx30*[*pmyo-3*::MYO-3::GFP; *pRF4*(*rol-6(su1006)*)]) and RB2525 (*C01G10.8(ok3501)*) were obtained from the *Caenorhabditis* Genetics Center. Animals were backcrossed to N2 at least four times. Strain ABZ100 [*unc-45(m94)*; *C01G10.8 (ok3501)*] was generated by crossing DR94 (*unc-45(m94)*) males with RB2525 (*C01G10.8 (ok3501)*) hermaphrodites. The *m94* allele was confirmed by incubating eggs at the restrictive temperature (25°C) and testing for the characteristic movement phenotype. The *ok3501* allele was confirmed by genotyping PCR (primers used: left primer AGAAACGGCGATACGGCTAA; right primer: CAATGCAAGCTCGCCCTATT).

Nematodes were grown on NGM plates seeded with the *Escherichia coli* OP50-1 strain at 15°C. Synchronized L1 larvae were obtained as follows: Synchronized high density plates containing adult worms and L1 progeny were washed with M9 solution and collected into tubes. Then, L1 larvae were separated from the adult worm population by centrifugation. For all experiments, 5–20 μl of M9 containing 30–130 L1 larvae where transferred onto fresh plates seeded with *E. coli* HT115 (DE3) containing the indicated RNAi vectors and grown at 15 or 20°C for the duration of the experiment. The animals were then taken for phenotypic analysis, imaging and/or antibody staining. At 15°C, the first day of egg-laying was set at 120 h after L1 transfer, at 65 h for growth at 20°C and at 50 h for growth at 25°C. The first day of egg-laying was defined as the point when animals grown on HT115 (DE3) bacteria containing an empty vector control were able to lay eggs.

### Ethyl methanesulfonate (EMS) screen

Thirteen thousand *unc-45(m94)* worms were grown at the permissive temperature (15°C) to young adulthood and mutagenized using 50 mM EMS, as described earlier (Brenner, 1974). To isolate both recessive and dominate suppressors, 88,000 F1 progeny were grown at the permissive temperature and F2 progeny were shifted to the restrictive temperature (22°C) at the L1 larvae stage, avoiding UNC-45-associated embryonic arrest. 18,000 viable F2 animals grown at the restrictive temperature were then gently transferred to new plates and placed distally from the food source. Seventy-seven animals that reached the food source and exhibited coordinated movement were individually transferred to new plates and phenotype transmission was determined at 20°C, with sterile lines being excluded. Next, the six remaining lines were assayed for motility (thrashing assay) and UNC-45 localization was imaged by immuno-staining with anti-UNC-45 antibodies (Gazda et al., 2013). Three lines that exhibited both normal motility and muscle organization, together with body wall muscle-localized UNC-45, were collected for sequencing analysis.

### RNA interference (RNAi)

RNAi knockdown treatments were performed as previously described (Ben-Zvi et al., 2009). Chaperone RNAi constructs were obtained from the “RNAi chaperone library” kindly provided by Prof. Richard Morimoto, Northwestern University. For all RNAi experiments, L1 larvae were obtained and transferred onto RNAi plates. Unless otherwise indicated, animals were maintained at 15°C as described above. Each RNAi treatment was performed at least three times, and >30 animals per experimental condition were scored. Animals fed on *E. coli* HT115 (DE3) containing the empty vector (pL4440) or *unc-45* RNAi were included as negative and positive controls in each experiment, respectively (Figure S1). In addition, changes in mRNA and encoded protein levels of several genes were determined by Real-Time PCR or Western blot, respectively, to test the efficiency of the RNAi treatment.

### Motility assays

Age-synchronized populations of >30 animals were used for each assay. Animals were grown at 15°C for the duration of the experiment. On the first and second days of egg-laying, worms were tested for paralysis/slow movement by striking the plates gently against a table three times and monitoring movement. Worms that moved their heads but failed to move their bodies were scored as paralyzed. RNAi knockdown of *unc-45* resulted in a strong paralyzed phenotype that was used as reference. To examine for small changes in motility, age-synchronized young adult animals (*n* = 20) movement was assessed by the thrashing assay, as previously described (Gazda et al., 2013).

### RNA levels

Total RNA was extracted from age-synchronized L4 animals using TRIzol reagent (Invitrogen). RNA was reverse transcribed using the iScript cDNA Synthesis Kit (Bio-Rad). mRNA levels were measured by quantitative PCR (qPCR), performed on a C1000 Thermal Cycler (Bio-Rad) with KAPA SYBRFAST qPCR Master Mix (KAPA Biosystems). The results were analyzed using CFX Manager software (Bio-Rad). Relative transcript levels were determined by averaging the C_T_ of triplicate values for the genes examined and normalizing those to C_T_ values obtained for 18S rRNA of the same sample using the 2^−ΔΔ*C*^_T_ method (Livak and Schmittgen, 2001).

### Protein levels

Age-synchronized populations were collected and lyzed in SDS sample buffer (98°C for 10 min). Samples were separated by SDS-SB gel electrophoresis and analyzed by western blot, using anti-tubulin (Sigma), anti-CeHop and anti-CeAha1 antibodies (kindly provided by Dr. Klaus Richter). Peroxidase-conjugated AffiniPure goat anti-rabbit antibodies served as secondary antibodies.

### Co-immunoprecipitation

An age-synchronized population was collected in M9 buffer, flash-frozen and lyzed using a hand drill. The lysate was maintained at 4°C. Samples were diluted in RIPA buffer (Sigma) supplemented with protease inhibitors (Roche) and centrifuged to remove debris. The supernatant was divided into equal aliquots. Two aliquots were incubated for 30 min at 4°C with either anti-CeHop or anti-CeAha1 antibodies and one aliquot was left untreated. Protein A/G PLUS-Agarose beads (Santa Cruz) were added to the samples, which were incubated for additional 30 min at 4°C. The samples were centrifuged (1000× g, 5 min) and supernatants and pellet were collected. Pellets containing the agarose beads were washed three times in RIPA buffer. Pellet and supernatant fractions were boiled (95°C for 10 min) in SDS sample buffer, resolved by SDS-SB gel electrophoresis and analyzed by western blot using anti-CeHop, anti-CeAha1, anti-CeHsp90 (kindly provided by Prof. Richard Morimoto) or anti-UNC-45 antibodies (Gazda et al., 2013). Peroxidase-conjugated AffiniPure goat anti-rabbit antibodies served as secondary antibodies.

### Immunofluorescence staining

Immunofluorescent staining of MYO-3, CeHop and CeAha1 was performed as previously described (Karady et al., 2013). Animals were stained with antibodies for MYO-3 (5-6-S, Hybridoma Bank), CeHop or CeAha1 (kindly provided by Dr. Klaus Richter). DyLight 488 goat anti-mouse, 549 goat anti-rabbit (Jackson Immuno-Research) or 633 goat anti-mouse (Thermo Scientific) antibodies served as secondary antibodies. Animals were imaged using a Leica DM5500 B confocal microscope. Fluorophores were excited using the 488, 549, and 633 nm laser lines, respectively. Immunofluorescent staining of UNC-45 was performed as described (Gazda et al., 2013).

## Results

### Using *unc-45* mutants to identify novel myosin-folding modifiers

**Figure 1:**
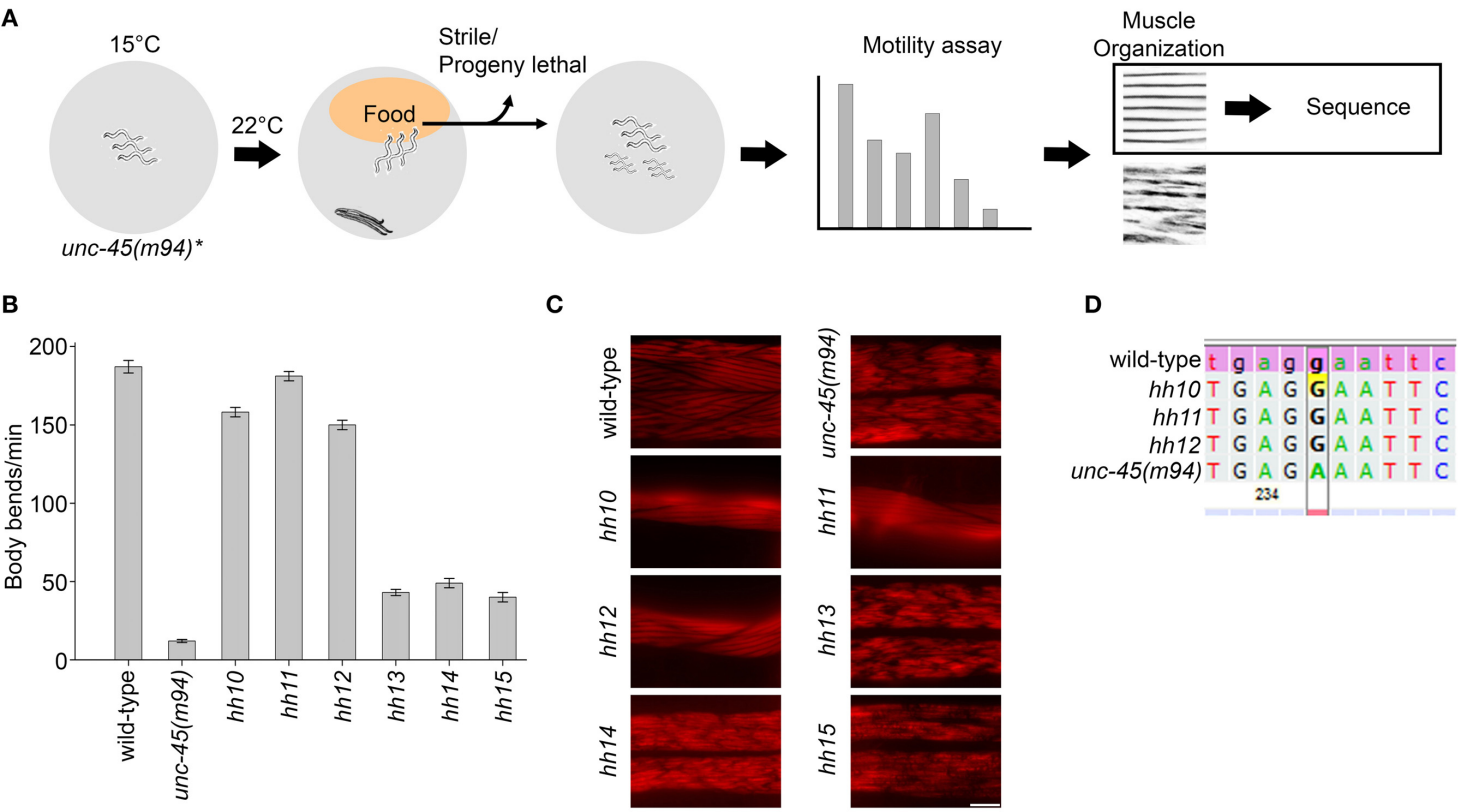
**EMS screen for suppressors of the *unc-45(m94)* phenotype under restrictive conditions. (A)** Schematic representation of the EMS-based screen used to identify suppressors of *unc-45(m94)* animals. **(B)** Age-synchronized wild-type, *unc-45(m94)* and isolated suppressor (*hh10*, *hh11*, *hh12*, *hh13*, *hh14*, and *hh15*) animals were grown at 22°C and scored for motility on day 1 of adulthood. Data are mean ± s.e.m. **(C)** Confocal images of body-wall muscle. Age-synchronized wild-type, *unc-45(m94)* and isolated suppressor (*hh10*, *hh11*, *hh12*, *hh13*, *hh14*, and *hh15*) animals were grown at 22°C, collected on day 1 of adulthood and stained with α-UNC-45 antibodies. Scale bar is 10 μm. **(D)** Sequence alignment of wild-type, *unc-45(m94)* and isolated suppressors (*hh10*, *hh11*, and *hh12*) highlighting base position 2341. Sequence analysis of selected suppressors (*hh10*, *hh11*, and *hh12*) revealed the A2341G mutation, which results in the synthesis of a wild-type version of the *unc-45* gene.

The gene encoding UNC-45, an essential muscle-specific chaperone, was used for our mutagenesis screening approach. A single amino acid substitute in the UCS domain of *C. elegans* UNC-45, E781K, or L822F (the *m94* and *e286* alleles, respectively), results in temperature-sensitive motility defects and myosin disorganization phenotypes when animals are grown at restrictive conditions (>22°C). However, animals show no phenotypes during development and early adulthood when grown at the permissive temperature (15°C). To identify chaperones that play a redundant role in myosin assembly and muscle maintenance, we screened for modifiers that can mask *unc-45*-associated phenotypes under restrictive conditions. Because RNAi knockdown of chaperone expression is expected to aggravate *unc-45* phenotypes, we used mutagenesis to screen for compensatory factors. Specifically, EMS was used to induce mutations in the germline of *unc-45(m94)* hermaphrodite worms. F2 animals were shifted to the restrictive temperature (22°C) and screened for mutants with improved motility. Mutant animals, identified by their ability to reach a distant *E. coli* food source on the plate, were isolated (Figure 1A). The identified suppressor mutants with restored UNC-45 muscle organization were selected for further genetic analysis (Figures 1A–C). We first sequenced the *unc-45* gene of the identified suppressors to search for possible intragenic changes. Of the three identified alleles (*hh10*, *hh11*, and *hh12*), all proved to be revertants, presenting the A2342G substitution that restores the *m94* allele of the *unc-45* gene to the wild-type version (Figure 1D). This observation suggests that introducing another change in the *unc-45* gene or a mutation(s) elsewhere in the genome was unable to compensate *unc-45(m94)*-specific phenotypes under restrictive conditions. This indicates an essential role for UNC-45 that cannot be bypassed by other chaperones.

### A targeted RNAi screen for chaperone function

**Figure 2:**
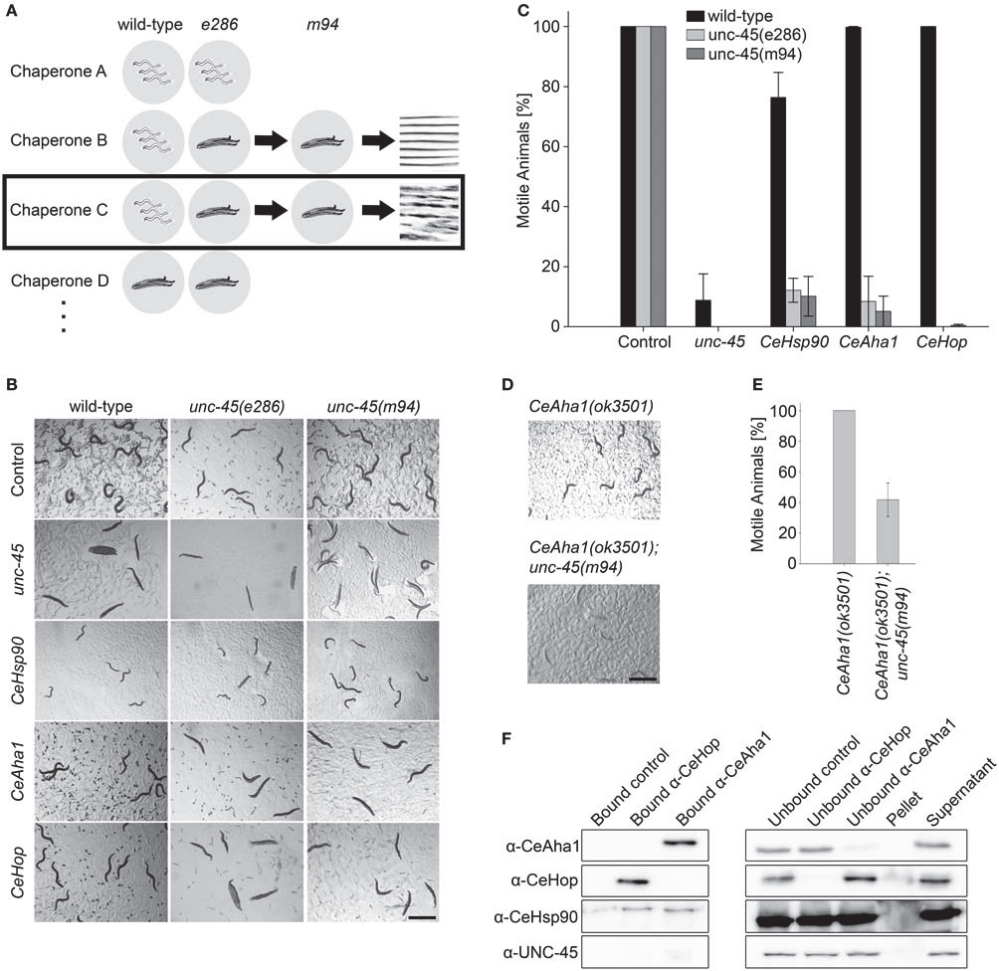
**Using *unc-45* as bait to identify novel myosin chaperones. (A)** Schematic representation of the *unc-45*-targeted screen. **(B)** Age-synchronized (L1) wild-type, *unc-45(e286)* or *unc-45(m94)* animals grown at 15°C were transferred to plates containing control, *unc-45*, *CeHsp90* (*daf-21*), *CeHop* (*sti-1*), or *CeAha1* (*C01G10.8*) RNAi-expressing bacteria and images were taken on day 2 of adulthood. Scale bar is 1 mm. **(C)** Age-synchronized (L1) wild-type, *unc-45(e286)* or *unc-45(m94)* animals treated as in (B) were scored for motility on day 2 of adulthood. Data are mean ± s.e.m. **(D)** Age-synchronized RB2525 [*C01G10.8(ok3501)*] or ABZ100 (*unc-45(m94)*; *C01G10.8(ok3501)*) animals were grown at 15°C on regular plates. Images were taken on day 2 of adulthood. Scale bar is 1 mm. **(E)** Age-synchronized (L1) RB2525 [*C01G10.8(ok3501)*] or ABZ100 [*unc-45(m94)*; *C01G10.8(ok3501)*] animals treated as in (D) were scored for motility on day 2 of adulthood. Data are mean ± s.e.m. **(F)** CeAha1, CeHop, Hsp90, or UNC-45 were co-immunoprecipitated from total extracts of wild-type animals using anti-CeHop or anti-CeAha1 antibodies. Non-specific binding (bound control), unbound fractions (unbound control, unbound Hop, and unbound Aha1), and untreated fractions (pellet and supernatant) are included for reference.

One possible interpretation of the mutagenesis screen results is that the lack of *unc-45(ts)* phenotypes at permissive conditions might result from the redundant but not compensatory functions of other chaperones in the muscle (Balch et al., 2008; Sawyer et al., 2011). We reasoned that affecting two chaperones that have no gross phenotypes on their own could counteract redundancy and uncover novel chaperone interactions. To test this approach, we first compiled a list of *C. elegans* chaperones (97 genes) (Table 1) and examined the effects of down-regulation of each chaperone in wild-type animals (Figure 2A). To draw up this list, we relied on gene annotations and biochemical and functional data. We focused our scope to specific chaperone families and their co-chaperones, including Hsp60 and Hsp10 (10 genes), Hsp70, Hsp40, and NEF (48 genes), Hsp90 and Hsp90 co-chaperones (21 genes) and sHSP (18 genes).

**Table 1:** **List of *C. elegans* chaperones and their associated phenotypes**.

	**Cosmid Name**	**Gene Name**	**Other Name**	**Phenotypes (RNAi exp)**	**Phenotypes (wormbase)**
Hsp60/Chaperonin	T05C12.7	*cct-1*	TRiC/CCT	Lva, Ste	Lva(1,3), Ste(3)
	T21B10.7	*cct-2*	TRiC/CCT	Lva, Ste	Lva(3), Ste(1,3)
	F54A3.3	*cct-3*	TRiC/CCT	Lva, Ste	Lva(3), Ste(1,5)
	K01C8.10	*cct-4*	TRiC/CCT	Lva, Ste	Lva(3), Ste(1,3)
	C07G2.3	*cct-5*	TRiC/CCT	Lva	Lva(1,3)
	F01F1.8	*cct-6*	TRiC/CCT	Lva, Ste	Lva(3), Ste/Let(2,3), Unc(3)
	T10B5.5	*cct-7*	TRiC/CCT	Lva, Ste	Lva(3), Ste(1)
	Y55F3AR.3	*cct-8*	TRiC/CCT	Lva, Ste	Lva(1,3), Ste(1), Unc(3)
	Y22D7AL.5	*hsp-60*	Hsp60	Lva, Ste	Lva(1), Ste(1)
	Y22D7AL.10		Hsp10		
Hsp70	F26D10.3	*hsp-1*	Hsc70	Lva, Ste, Unc	Lva(1,3), Ste/Let(2,3), Unc(3)
	F11F1.1		Hsc70		
	C15H9.6	*hsp-3*	BIP	-	Lva(3), Ste(3), Unc(3)
	F43E2.8	*hsp-4*	BIP	Lva, Ste	Lva(1,3), Ste(1)
	F54C9.2	*stc-1*	STCH	-	Lva(3), Unc(1,3)
	C37H5.8	*hsp-6*	Mortalin	Lva	Lva(1,3), Ste/Let(2,3)
	C12C8.1	*hsp-70*	Hsp70	-	-
	F44E5.5		Hsp70	-	-
	F44E5.4		Hsp70	-	-
Hsp40/DNAJ	B0035.14	*dnj-1*		-	-
	B0035.2	*dnj-2*		-	-
	C01G10.12	*dnj-3*		-	Ste/Let(2)
	C01G8.4	*dnj-4*		-	Ste/Let(2)
	C04A2.7	*dnj-5*		-	-
	C55B6.2	*dnj-7*		-	-
	C56C10.13	*dnj-8*		-	Ste/Let (2), Unc(3)
	F11G11.7	*dnj-9*		-	-
	F22B7.5	*dnj-10*		-	Lva(3), Let/Ste(2)
	F38A5.13	*dnj-11*		-	Let/Ste(2)
	F39B2.10	*dnj-12*		-	Let/Ste(2)
	F54D5.8	*dnj-13*		-	-
	K02G10.8	*dnj-14*		-	-
	K08D10.2	*dnj-15*		-	Ste/Let(2)
	R74.4	*dnj-16*		-	-
	T03F6.2	*dnj-17*		-	-
	T04A8.9	*dnj-18*		-	Ste/Let(2)
	T05C3.5	*dnj-19*		-	-
	T15H9.7	*dnj-20*		-	-
	T19B4.4	*dnj-21*		-	Lva(3), Ste(3)
	T23B12.7	*dnj-22*		-	Lva(3), Ste/Let(2,3), Unc(1,3)
	T24H10.3	*dnj-23*		-	Unc(3)
	W03A5.7	*dnj-24*		-	Ste/Let(2)
	W07A8.3	*dnj-25*	Auxilin	-	Lva(3)
	Y39C12A.8	*dnj-26*		-	-
	Y47H9C.5	*dnj-27*		-	-
	Y54E10BL.4	*dnj-28*		-	-
	Y63D3A.6	*dnj-29*	SEC63	-	Lva(1,3), Ste/Let(2)
	Y71F9B.16	*dnj-30*		-	-
	F18C12.2	*rme-8*	RME8	-	Lva(3), Unc(1,3), Ste(3), Let(3)
	Y39C12A.9			-	-
	K07F5.16			-	-
	F54F2.9			-	-
NEF	F57B10.11	*bag-1*	BAG1	-	-
	H14N18.1	*unc-23*	BAG2	Unc	Unc(1,3)
	C34C12.8		GrpE	-	Lva(1,3), Let(1)
	C30C11.4	*hsp-110*	Hsp110	-	Unc(3)
	T24H7.2		Hsp110	-	-
	T14G8.3		Hsp110	-	-
Hsp90	C47E8.5	*daf-21*	Hsp90	Lva, Ste	Lva(1,3), Ste/Let(2)
	T05E11.3	*enpl-1*	GRP94	-	Lva(3), Ste(3), Ste/Let (2), Unc(3)
	R151.7	*trap-1*	mtHsp90	-	-
Hsp90 co-chaperones	C01G10.8		AHA1	-	-
	R09E12.3	*sti-1*	HOP/STI1	-	Ste(3)
	ZC395.10		P23	-	-
	F31D4.3	*fkb-6*	FKBP	Lva	Ste(2)
	W08F4.8	*cdc-37*	CDC37	Lva	Ste/Let(2,3), Unc(1,3)
	T09B4.10	*chn-1*	CHIP	-	-
	Y39B6A.2	*pph-5*	PP5	-	-
	R05F9.10	*sgt-1*	SGT	-	Lva(3)
	F30H5.1	*Unc-45*	UNC45	Ste, Unc	Ste/Let(1,2), Unc(1,3)
	ZK370.8		TOM70	-	-
	Y22D7AL.9			Lva	-
	T12D8.8	*hip-1*	ST13	-	-
	C56C10.10		AIP1	Lva	-
	C34B2.5		TTC1	-	Lva(1)
	C33H5.8		RAPAP3	-	-
	C17G10.2		TTC4	-	Let(1), Unc(1,3), Ste(3)
	C17G10.10		CNS1	-	-
	D1054.3		SGT	Not tested**	Let(2)
sHSP	T22A3.2	*hsp-12.1*		-	-
	C14B9.1	*hsp-12.2*		-	Ste(3)
	F38E11.1	*hsp-12.3*		-	-
	F38E11.2	*hsp-12.6*		-	-
	T27E4.8	*hsp-16.1*		-	-
	T27E4.2	*hsp-16.11*		-	-
	Y46H3A.3	*hsp-16.2*		-	-
	Y46H3A.2	*hsp-16.41*		-	-
	T27E4.3	*hsp-16.48*		-	-
	T27E4.9	*hsp-16.49*		-	-
	F52E1.7	*hsp-17*		-	-
	C09B8.6	*hsp-25*		-	-
	C14F11.5	*hsp-43*		-	-
	F43D9.4	*sip-1*		-	-
	ZK1128.7			-	-
	Y55F3BR.6			-	-
	F08H9.4			-	-
	F08H9.3			-	-

A list of C. elegans chaperones (97 genes), including Hsp60 and Hsp10 (10 genes), Hsp70, Hsp40, and NEF (48 genes), Hsp90 and Hsp90 co-chaperones (21 genes) and sHSP (18 genes). For each chaperone C. elegans cosmid and gene names and other names (if applicable) are listed. RNAi was introduced at L1 and phenotypes (Larval arrest, Lva; Sterility, Ste; Larval lethality, Let or strong Uncoordination, Unc) were scored after 5 days at 15 °C or after 3 days at 20 °C. For each chaperone, annotated wormbase (www.wormbase.org) phenotypes (Lva; Ste; Let; or Unc) are listed. (1) Denote phenotypes listed for wild-type RNAi treatment. (2) Denote phenotypes listed for mutant animals. (3) Denote phenotypes listed for RNAi-sensitized background or treatments. We defined a phenotype as more than ~70% of the animals showing the phenotype in three independent experiments. ^**^D1054.3/SGT was identified as a Hsp90 co-chaperone after the screen was performed (Eckl et al., 2014).

RNAi was introduced at the first larval stage of the *C. elegans* life cycle (L1) and its effects were examined after 5 days at 15°C (or after 3 days at 20°C), until animals grown on control RNAi reached adulthood (Table 1). Down-regulation of genes encoding 10 chaperones (17 genes), including CTT, HSP-1 (Hsc70/HSPA8), HSP-4 (BIP), HSP-6 (Mortalin), and DAF-21 (Hsp90), resulted in severe organismal phenotypes, such as lethality, developmental arrest, or sterility (Table 1, Figure S2). In contrast, down-regulation of genes encoding most chaperones examined had no apparent phenotypes (Table 1). Our data are in agreement with published RNAi screens for wild-type RNAi treatment (Table 1, data curated by wormbase). When knockout mutants or data from enhanced RNAi efficacy experiments, (for example via dsDNA injection into the gonads) were considered, more acute phenotypes were observed for some chaperones (Table 1, data curated by wormbase). This further supports our conclusion that the proteostasis network may buffer mild perturbations in the folding capacity of the organism.

As expected, down-regulation of only two chaperones, *unc-45* or *unc-23*, showed a marked movement defect. For example, based on their impact on muscular ultrastructure or their involvement in myopathies, molecular chaperones, such as TRIC (*cct*), αB-crystallin/HSPB5 (*hsp-25*), and DNAJB6 (*dnj-24*), were suggested to play a key role in sarcomere assembly and maintenance (Ding and Candido, 2000; Vang et al., 2005; Kim et al., 2008; Willis et al., 2009; Willis and Patterson, 2010; Garrido et al., 2012; Haslbeck et al., 2012; Sarparanta et al., 2012). Although these chaperones are associated with the sarcomere (Leroux and Candido, 1997; Ding and Candido, 2000; Meissner et al., 2011), knockdown of their genes by feeding RNAi did not result in notable motility defects (Table 1).

### Screening for chaperones that genetically interact with *unc-45* mutants

RNAi knockdown of chaperones was next performed with *unc-45(ts)* mutant strains under permissive conditions. Age-synchronized L1 *unc-45(e286)* mutant animals kept at permissive conditions (15°C) were treated with RNAi for different molecular chaperones and monitored for motility defects. We first sought chaperones that cause *unc-45(e286)*-specific phenotypes at permissive conditions when depleted (Figure 2A). Of the 96 RNAi constructs tested, five genes induced loss of coordination and/or an egg-laying defect, resulting in reduced motility in more than 70% of the *unc-45(e286)* mutant animals. As noted above, down-regulation of *unc-45* or *unc-23* also induced a loss of coordination in wild-type animals (Table 1); these were discarded from the candidate gene list (Figure 2A). The remaining chaperones identified in the screen are *CeHsp90 (daf-21)*, *CeHop (sti-1)* and *CeAha1 (C01G10.8)* (Figures 2B,C). RNAi treatment for *CeHsp90*, encoding a known myosin chaperone, resulted in a strong larval arrest phenotype in both wild-type and *unc-45(e286)* animals but induced a strong motility defect only in *unc-45(e286)* animals (76.4 ± 8.3%, as compared to 12.1 ± 4% motile animals). *CeHop* and *CeAha1* RNAi only affected *unc-45(e286)*-expressing animals, where complete paralysis resulted (Figures 2B,C).

Given that genetic background variations can confound the interpretation of genetic interactions, the three genes that affected *unc-45(e286)* were tested for their effects on animals carrying the *unc-45(m94)* allele (Figure 2A). Age-synchronized L1 *unc-45(m94)* mutant worms maintained under permissive conditions were treated with RNAi for *CeHsp90*, *CeHop* or *CeAha1* and compared to animals that took up the empty vector control. Similar to the *e286* allele, *unc-45(m94)* animals treated by RNAi for *CeHsp90*, *CeHop*, or *CeAha1* were completely paralyzed on day 2 of adulthood under permissive conditions (Figures 2B,C). Although *CeAha1* or *CeHop* RNAi treatment reduced the levels of the corresponding proteins to a similar extent in all strains examined, *CeHop* and *CeAha1* RNAi only induced paralysis in *unc-45(ts)*-expressing animals (Figures 2B,C and Figure S3). Moreover, only 41.8 ± 11% of the double mutant of *unc-45(m94)* and a deletion allele of *CeAha1* (RB2525 strain) were motile by the second day of adulthood, in contrast to the respective single mutant strains (Figures 2D,E). Thus, *CeHop* and *CeAha1* are novel genetic modifiers of *unc-45* and are associated with muscle function.

Hop/STI1 (Hsp70-Hsp90 Organizing Protein/STress Inducible 1) and Aha1 (Activator of Hsp90 ATPase 1) are co-chaperones of Hsp90 that facilitate the maturation of Hsp90 substrates by modulating the Hsp90 ATPase cycle and thus driving Hsp90 conformational changes (Rohl et al., 2013). Hop/STI1 and Aha1 homologs in *C. elegans* were identified based on sequence analysis (Haslbeck et al., 2012). *CeHop* was shown to interact with Hsp90 by immunoprecipitation and to inhibit Hsp90 ATPase *in vitro*, similar to yeast Sti1 (Gaiser et al., 2009; Song et al., 2009). It is, therefore, likely that the interactions of *CeHop* and *CeAha1* with *unc-45* are mediated via CeHsp90. To test this directly, we performed immunoprecipitation and western blot analysis to monitor whether CeHop and CeAha1 form complexes with UNC-45 and CeHsp90. CeHop was found to be associated with CeHsp90 but not with CeAha1 or UNC-45 in agreement with previous mass spectrometry and co-immunoprecipitation studies (Song et al., 2009). Likewise, pull down of CeAha1 from wild-type total lysates captured CeHsp90 but not CeHop or UNC-45 (Figure 2F). Thus, as expected from their roles in other systems (Rohl et al., 2013), CeAha1 and CeHop form discrete complexes with CeHsp90 and do not directly interact with UNC-45.

### The Hsp90 co-chaperones CeHop and CeAha1 are linked to muscle homeostasis

**Figure 3:**
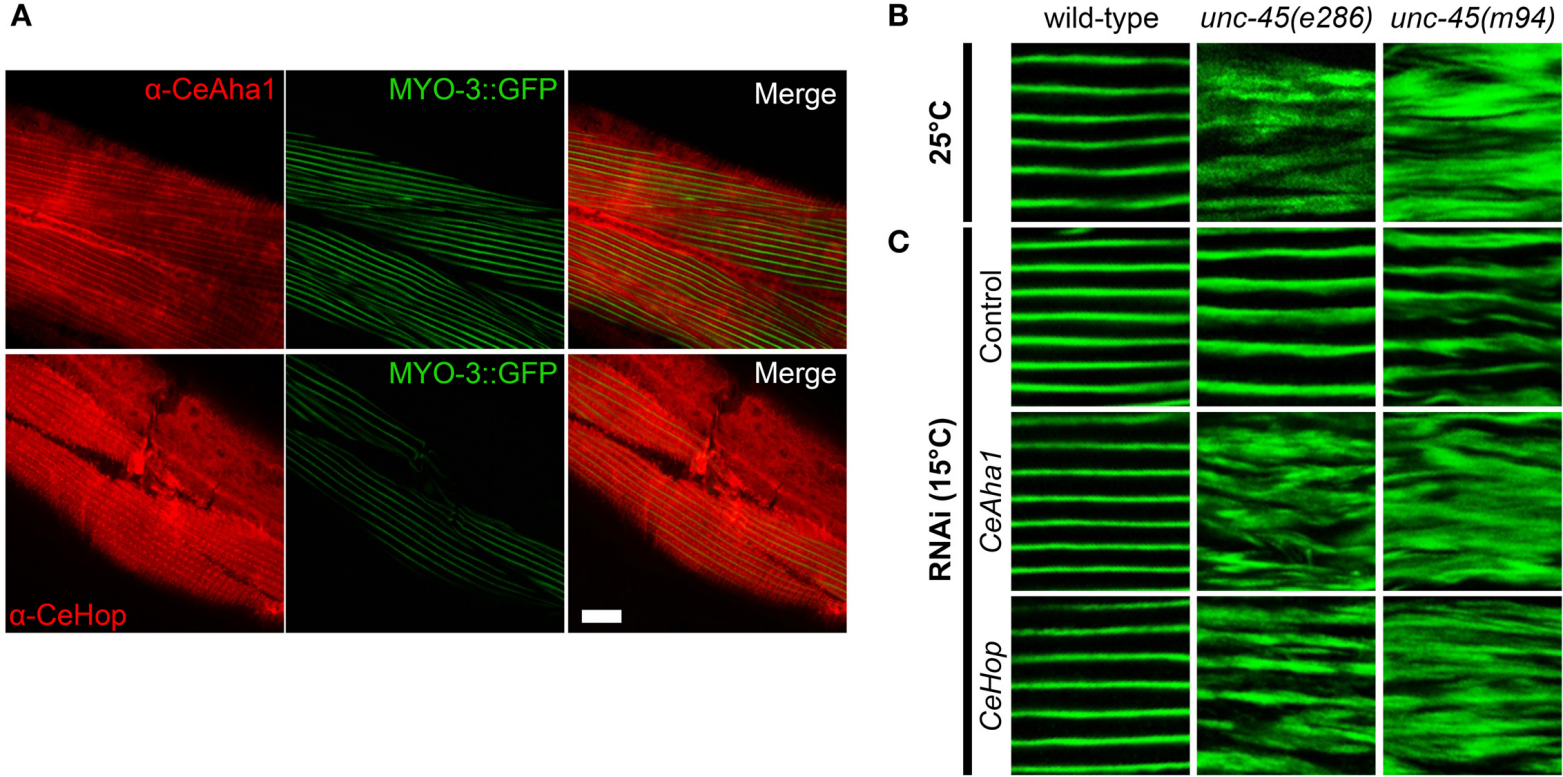
**CeAha1 and CeHop are novel modifiers of *unc-45* function**. **(A)** Age-synchronized (L1) RW1596 animals, expressing MYO-3:GFP, were grown at 15°C, collected on day 2 of adulthood, stained with specific antibodies to CeAha1 or CeHop, and imaged by confocal microscopy. Scale bar is 10 μm. **(B)** Confocal images of body wall muscle. Age-synchronized wild-type, *unc-45(e286)* or *unc-45(m94)* animals were grown at 25°C, collected on day 2 of adulthood and stained with anti-MYO-3 antibodies. Scale bar is 10 μm. **(C)** Confocal images of body-wall muscle. Age-synchronized wild-type, *unc-45(e286)* or *unc-45(m94)* animals were grown at 15°C, transferred to plates containing control, *CeAha1* (*C01G10.8*) or *CeHop* (*sti-1*) RNAi-expressing bacteria, collected on day 2 of adulthood and stained with anti-MYO-3 antibodies. Scale bar is 10 μm.

CeHsp90 is a well-established myosin chaperone (Barral et al., 2002; Etard et al., 2007; Gazda et al., 2013), is localized to sarcomeric structures and affects myofilament organization in body wall muscles of *C. elegans* (Gaiser et al., 2011). To determine whether CeHop and CeAha1 also affect myosin folding, we first examined whether these proteins are also localized to the sarcomere. Immunofluorescent staining using specific antibodies to CeHop and CeAha1 revealed striated patterns, suggesting a role for these proteins in myofilament formation and/or maintenance (Figure 3A).

To directly test this prediction, we examined whether *CeHop*- and *CeAha1*-associated *unc-45* phenotypes display disrupted myofilament organization. Therefore, we monitored the subcellular organization of myosin heavy chain A (MYO-3) using immuno-staining assays. Animals expressing *unc-45(e286)* or *unc-45(m94)* that were shifted to restrictive conditions after the first larval stage exhibited disruption of myofilament structure associated with mislocalization of MYO-3, while wild-type animals were unaffected (Figure 3B). MYO-3 organization in wild-type, *unc-45(e286)*, or *unc-45(m94)* mutant animals under permissive conditions was mostly unaffected by the third day of adulthood, although myosin disorganization was observed at later time points (Ben-Zvi et al., 2009). Treatment of wild-type animals with *CeHop* and *CeAha1* RNAi did not affect myofilament organization (monitored until the second day of adulthood). In contrast, treatment of *unc-45(e286)* mutant animals with *CeHop* and *CeAha1* RNAi resulted in complete disruption of myofilament organization and MYO-3 mislocalization under permissive conditions (Figure 3C). Similar results were observed for *unc-45(m94)*, although this mutant showed mild myosin disorganization in control animals. *CeHop* or *CeAha1* RNAi-induced myofilament disruption was similar to the MYO-3 disorganization observed when *unc-45* mutant animals were shifted to restrictive conditions (Figure 3B). Taken together, these data suggest that the Hsp90 co-chaperones CeHop and CeAha1 affect myosin organization when UNC-45 function is reduced. Moreover, the data suggest that Hsp90 and its co-chaperones are required to maintain *unc-45(e286)* and *unc-45(m94)* muscle organization.

### The Hsp90 co-chaperone Cep23 (ZC395.10) is required for myosin folding

**Figure 4:**
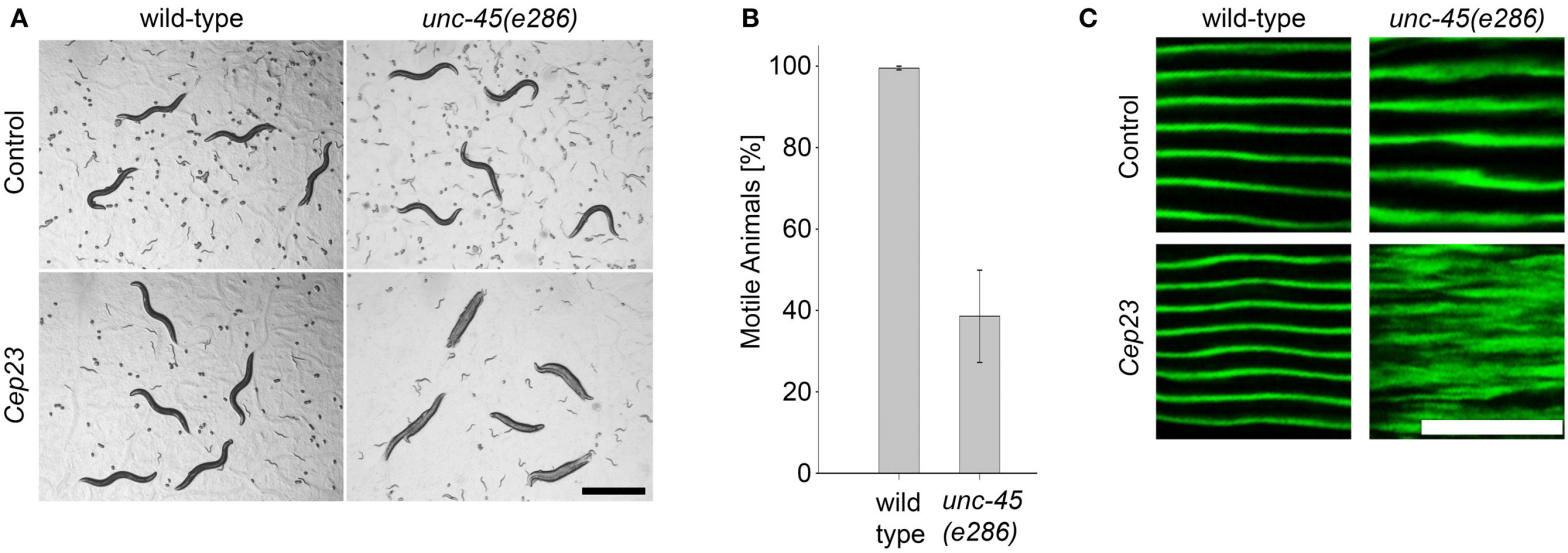
**The Hsp90 co-chaperone Cep23 is required for myosin folding. (A)** Age-synchronized (L1) wild-type or *unc-45(e286)* animals grown at 15°C were transferred to control or *Cep23* RNAi-expressing bacteria and imaged on day 2 of adulthood. Scale bar is 1 mm. **(B)** Age-synchronized (L1) wild-type animals treated as in (A) were scored for motility on day 2 of adulthood. Data are mean ± s.e.m. **(C)** Confocal images of body wall muscle. Age-synchronized wild-type or *unc-45(e286)* mutant animals were grown at 15°C, transferred to plates containing control or *Cep23* (*ZC395.10*) RNAi-expressing bacteria, collected on day 2 of adulthood and stained with anti-MYO-3 antibodies. Scale bar is 10 μm.

The specific role of the Hsp90 system in modulating UNC-45-associated phenotypes suggested that other Hsp90 co-chaperones not identified in our screen might be required for *unc-45(ts)* motility. To re-examine the role of these co-chaperones in muscle function, wild-type and *unc-45(e286)* animals were treated with RNAi for known Hsp90 co-chaperones (Haslbeck et al., 2012, 2013) (17 genes; Table 1) and changes in motility were monitored. The detection threshold was increased in this secondary screen and animals were scored for any change in motility. Of the genes examined, only down-regulation of *C. elegans* p23 (*Cep23*; *ZC395.10*) resulted in motility defects of *unc-45(e286)* mutant animals but not wild-type animals. 39 ± 11.3% of the *unc-45(e286)* mutant animals examined were motile (Figures 4A,B and data not shown). Although *Cep23* modulated *unc-45(ts)* motility to a lesser extent than *CeHop* or *CeAha1* (Figures 2C, 4B), *Cep23* effect on *unc-45(ts)* motility was highly significant (*p* < 0.005). *Cep23* was considered as false negative of the screen and its role as an UNC-45 modifier was next examined.

The Hsp90 co-chaperone p23 is an important component of the general Hsp90 chaperone cycle, forming a complex with CeHsp90 (Gaiser et al., 2010) that is localized to the sarcomere (Meissner et al., 2011). To test whether *Cep23(RNAi)* also affected myofilament organization, wild-type and *unc-45(e286)* mutant animals were monitored for changes in the subcellular organization of MYO-3 using immuno-staining assays. In agreement with the *Cep23*-mediated effects on motility, treatment of *unc-45(e286)* animals with *Cep23(RNAi)* resulted in severe MYO-3 disruption in affected animals (Figure 4C). Thus, *Cep23* genetically interacts with *unc-45*, further supporting the specific role of the Hsp90 chaperone system in modulating *unc-45* phenotypes.

### Using novel chaperone interactions in muscle to extend the sarcomere quality control network

**Figure 5:**
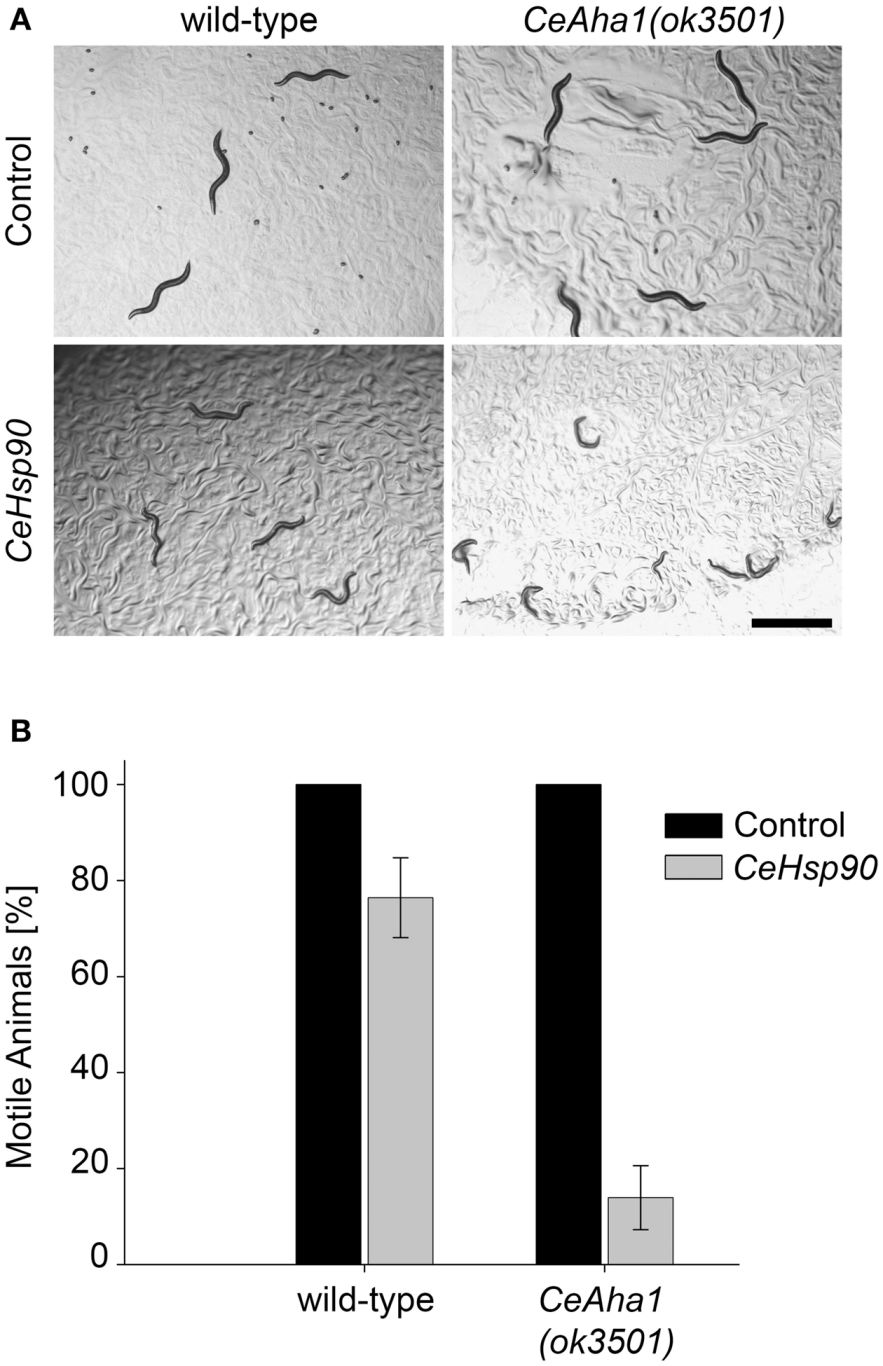
**Extending the UNC-45 interaction network. (A)** Age-synchronized (L1) wild-type or RB2525 [*C01G10.8(ok3501)*] animals grown at 15°C were transferred to plates containing control or *CeHsp90* (*daf-21*) RNAi-expressing bacteria and images were taken on day 2 of adulthood. Scale bar is 1 mm. **(B)** Age-synchronized (L1) wild-type or RB2525 [*C01G10.8(ok3501)*] animals, treated as in (A), were scored for motility on day 2 of adulthood. Data are mean ± s.e.m.

To further extend the network of chaperones associated with muscle function, the chaperones identified in the screen can, in turn, be used to uncover novel chaperone interactions. As a proof of concept, the motility of *CeAha1* mutant animals was used as bait to reveal interactions of this gene with *CeHsp90* in muscle function. When *CeAha1(ok3501)* knockout animals were treated with *CeHsp90(RNAi)*, only 13.9 ± 7% of the animals were motile by the first day of egg-laying, while no effect on motility was observed for *CeAha1(ok3501)* animals grown on the empty vector control (Figures 5A,B). This was specific to *CeAha1(ok3501)* animals, as most of the wild-type animals treated with CeHsp90 RNAi were motile (76.6±4%) (Figure 5B). Moreover, both wild-type and *CeAha1(ok3501)* animals treated with *CeHsp90(RNAi)* showed a complete larval arrest phenotype (Figure 5A). Thus, using *CeAha1* as bait uncovered its interactions with *CeHsp90* in muscle function and may serve to further uncover novel interactions with other molecular chaperones.

## Discussion

### CeHop, CeAha1 and Cep23 are novel myosin co-chaperones

Incorrect folding and assembly of the sarcomere are prominent contributors to disease and aging (Herndon et al., 2002; Benian and Epstein, 2011). There is only limited understanding of the chaperone networks involved in sarcomere assembly and maintenance. Here, we used a genetic interaction approach as applied to a *C. elegans* body wall muscle model to identify novel chaperones that are required for sarcomere assembly and maintenance. To screen for novel sarcomere-associated chaperones, we specifically challenged the proteostasis network by studying animals with mutations affecting a known muscle chaperone, *unc-45*. These unbiased screens were able to genetically verify the known UNC-45-Hsp90 interaction (Barral et al., 2002; Gazda et al., 2013) and identified three Hsp90 co-chaperones, CeHop (STI-1), CeAha1 (C01G10.8) and Cep23 (ZC395.10), not previously associated with myosin folding (Gaiser et al., 2009, 2010, 2011; Song et al., 2009; Meissner et al., 2011; Haslbeck et al., 2012). Hsp90 binds numerous co-chaperones (17 in *C. elegans*; Table 1) (Haslbeck et al., 2012). Thus, our screen identified a specific set of co-chaperones required for Hsp90-myosin folding, including a client recruiter co-chaperone, CeHop, a remodeling co-chaperone, CeAha1, and a client maturation co-chaperone, Cep23. This set of co-chaperones forms a core chaperone folding cycle (Rohl et al., 2013). The screen, therefore, highlights the Hsp90 ATPase cycle as critical for Hsp90 function in myosin folding, specifically when UNC-45 function is compromised.

### Mild perturbations of the proteostasis network uncover specific chaperone interactions

Down-regulation of chaperone expression in an *unc-45* mutant background did not lead to general proteostasis sensitivity as reflected by strong motility defects, regardless of the individual chaperone down-regulated. Rather, a specific set of chaperones was identified that function together to modulate Hsp90 substrate binding, remolding and releasing, and that buffer myosin folding when UNC-45 function is perturbed. This finding is strengthened by the *unc-45(m94)*-based mutagenesis screen, which only identified *unc-45(m94)* revertants as modifiers of UNC-45 function under restrictive conditions. In agreement, epistatic analyses of yeast genes required for folding in the endoplasmic reticulum identified specific aggravating and improving interactions between molecular chaperones (Jonikas et al., 2009), suggesting that mild perturbation of the proteostasis network can uncover specific chaperone interactions. Moreover, the discovery of a specific set of chaperones, such as *unc-45* modulators, suggests that the proteostasis buffering capacity depends on modulating the function of chaperones that cooperate in the same process. In agreement, DnaK and trigger factor, chaperones that overlap in binding newly synthesized proteins, are redundant for bacterial growth under non-stress conditions but become essential when both are affected (Deuerling et al., 1999; Teter et al., 1999). Thus, a combination of mild perturbations of the proteostasis network, as when mildly affecting two chaperones, are required to uncover novel and specific chaperone interactions.

This specificity is somewhat surprising given that the chronic expression of misfolded proteins or aging unmask many unrelated metastable proteins (Gidalevitz et al., 2006; Ben-Zvi et al., 2009; Shemesh et al., 2013). The difference between the impact we observed for UNC-45 and the effect of aggregation-prone proteins could result from the impact of misfolded proteins and age-dependent modulation of the proteome on the proteostatic network (Shai et al., 2014). Indeed, expression of three distinct SOD1 mutants in *C. elegans* body wall muscle cells resulted in differential exposure of metastable mutant phenotypes, suggesting that each SOD1 mutant affects the proteostasis network differently (Gidalevitz et al., 2009). Moreover, expression of disease-related proteins was shown to inhibit calthris-mediated endocytosis in mammalian cells by engaging Hsc70. While over-expression of Hsc70 restored calthris-mediated endocytosis, it did not suppress aggregation (Yu et al., 2014). Given that other chaperones, such as Hsp90, DNJA, and Hsp110, were found to be associated with aggregates (Olzscha et al., 2011; Kirstein-Miles et al., 2013), many downstream functions may be affected by the expression of chronically misfolded proteins, resulting in a serious perturbation of the proteostatic network. This is in agreement with the organismal effects we noted upon down-regulating main folding chaperones, such as Hsc70 and mortalin (Figure S2). Indeed, more acute phenotypes were observed for many chaperones when knockout mutants are examined or when RNAi penetrance or efficacy was enhanced (Table 1). The enhanced penetrance of the phenotype could result from further reduction in the levels of expression or function of the chaperones, inducing more severe damage that can lead to different chaperonopathies, such as mutations in DNAJB6 that cause limb-girdle muscular dystrophy (Sarparanta et al., 2012). Thus, gross perturbations of the proteostatic network, as occur, for example, upon expression of aggregation-prone proteins, following down-regulation of a set of chaperones or with age, mask specific interactions and result in a failure of the quality control system to maintain proteostasis.

### A substrate-centric chaperone interaction map

One possible interpretation of our data is that different co-chaperones form functional modules *in vivo* that are specialized for a set of substrates to specifically impact their folding. This view of functional modules within the proteostasis network is supported by the roles of co-chaperones in directing chaperone function. For example, Hsp90 has different co-chaperones that perform similar functions for different substrates. Accordingly, kinases and steroid receptors interact with different subsets of Hsp90 co-chaperones (Rohl et al., 2013). Likewise, the combination of J proteins and NEF varies as a function of substrate and cellular function (Kampinga and Craig, 2010; Abrams et al., 2014). This view is further supported by our finding that *C. elegans* is more sensitive to down-regulation of genes encoding ATPase chaperone machines than those encoding their co-chaperones (compare 13/21 Hsp60/Hsp70/Hsp90 genes to 4/58 Hsp60/Hsp70/Hsp90 co-chaperone genes, Table 1). Co-chaperones that adjust chaperone function to a specific substrate demands may, therefore, give rise to a hierarchical structure of the proteostasis network with partially independent substrate-specific modules.

By directing our examination to how chaperones orchestrate sarcomere assembly and maintenance, we were able to identify key players in sarcomere folding that safeguard the functionality of muscle cells and are at risk in disease and aging. Any newly identified chaperone can be used to find additional chaperones and/or co-factors associated with muscle folding and assembly. Focusing on the substrate, rather than the chaperone, helps in the drawing of a genetic interaction map describing chaperone-substrate interactions *in vivo*.

### Conflict of interest statement

The authors declare that the research was conducted in the absence of any commercial or financial relationships that could be construed as a potential conflict of interest.
