# Supplementary material for: Challenging muscle homeostasis uncovers novel chaperone interactions in Caenorhabditis elegans
Source: Front Mol Biosci. 2014 Nov 6;1:21. doi: 10.3389/fmolb.2014.00021 (PMC4428482; doi:10.3389/fmolb.2014.00021)
Supplement: Supplementary file 1 [file Image1.PDF]

Supplementary Material

Challenging muscle homeostasis uncovers novel chaperone interactions in *Caenorhabditis elegans*

Anna Frumkin^[1], Shiran Dror^[1], Wojciech Pokrzywa[2,3], Yael Bar-Lavan[1], Ido Karady[1], Thorsten Hoppe[2,3], Anat Ben-Zvi[1]*

[1]Department of Life Sciences and The National Institute for Biotechnology in the Negev, Ben-Gurion University of the Negev, Beer Sheva, Israel
[2]Institute for Genetics, University of Cologne, Cologne, Germany
[3]Cologne Excellence Cluster on Cellular Stress Responses in Aging-Associated Diseases (CECAD), CECAD Research Center, University of Cologne, Cologne, Germany

*** Correspondence:** Dr. Anat Ben-Zvi, Department of Life Sciences, , Ben-Gurion University of the Negev, P.O. Box 653, Beer Sheva, , 84105, Israel.
anatbz@bgu.ac.il

1. Supplementary Figures

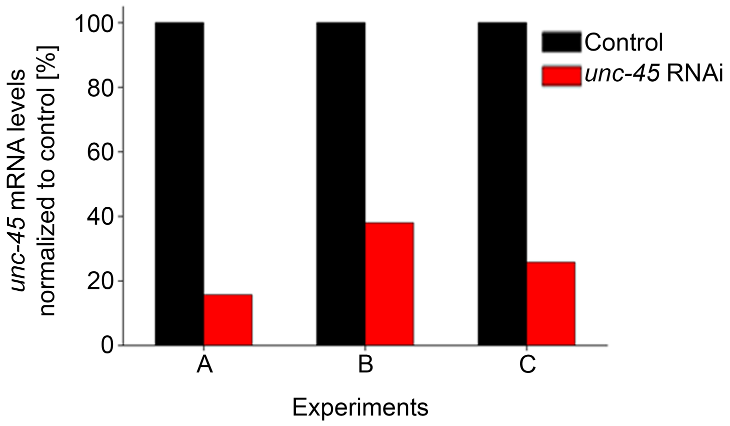

Supplementary Figure 1. Negative and positive controls used in RNAi experiments. Age-synchronized (L1) wild-type animals grown at 15°C were transferred to plates containing control or *unc-45* RNAi-expressing bacteria and *unc-45* mRNA levels were determined using qPCR. mRNA levels were normalized to control in each experiment.

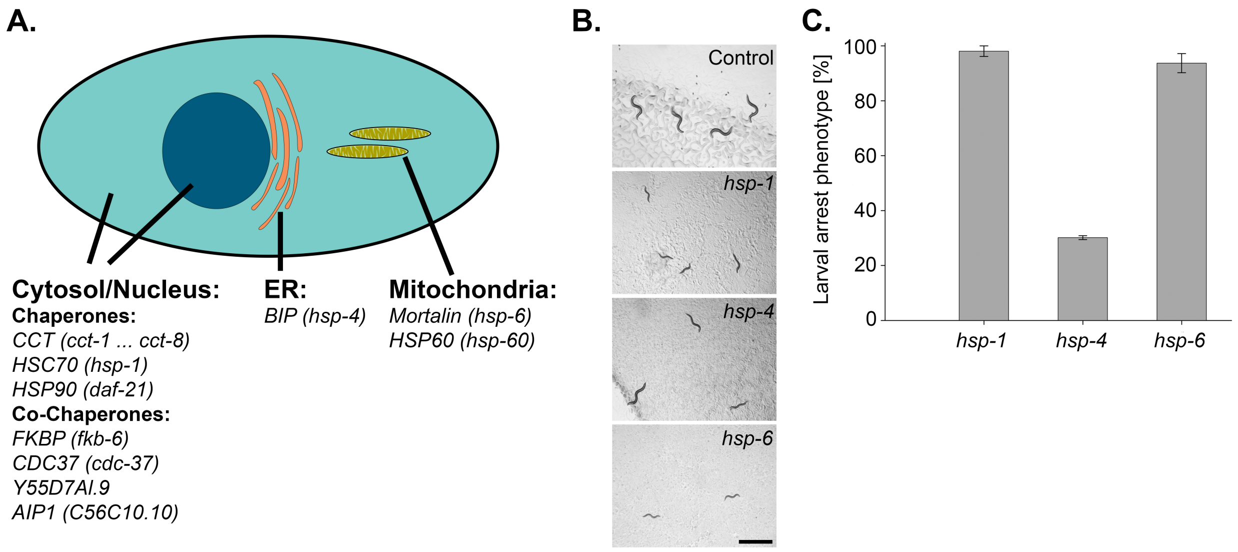

Supplementary Figure 2. RNAi-based down-regulation of the expression of most ATP-fuelled chaperones results in strong organismal phenotypes. (A) List of chaperones which cause severe organismal phenotypes when down-regulated, sorted by compartmental localization. (B) Age-synchronized (L1) wild-type animals grown at 15°C were transferred to plates containing control, *hsp-1*, *hsp-4* or *hsp-6* RNAi-expressing bacteria and images were taken on day 1 of adulthood. Scale bar is 1 mm. (C) Age-synchronized (L1) wild-type animals treated as in (B) were scored for paralysis on day 1 of adulthood. Data are mean ± SEM.

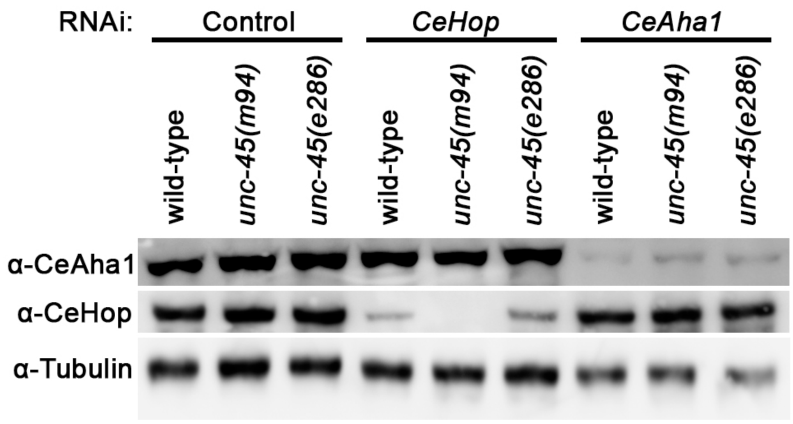

Supplementary Figure 3. CeHop and CeAha1 protein levels were monitored following RNAi treatment. Age-synchronized (L1) wild-type, *unc-45(e286)* or *unc-45(m94)* animals grown at 15°C were transferred to plates containing control, *CeHop* or *Ce Aha1* RNAi-expressing bacteria and protein levels were analyzed by western blot using CeAha1, CeHop or Tubulin antibodies.
